# Burden of disease and associated complications of hepatitis a in children and adults in Mexico: A retrospective database study

**DOI:** 10.1371/journal.pone.0268469

**Published:** 2022-05-18

**Authors:** Adriana Guzman-Holst, Gerardo Luna-Casas, Ana Burguete Garcia, Vicente Madrid-Marina, Maria Yolanda Cervantes-Apolinar, Anar Andani, Gloria Huerta-Garcia, Gilberto Sánchez-González

**Affiliations:** 1 GSK, Panama, Panama; 2 Estimatio SC, Mexico City, Mexico; 3 Instituto Nacional de Salud Pública (INSP), Cuernavaca, Mexico; 4 GSK, Mexico City, Mexico; 5 GSK, Wavre, Belgium; Centers for Disease Control and Prevention, UNITED STATES

## Abstract

**Background:**

Hepatitis A virus (HAV) infection is a leading cause of viral hepatitis in children, yet the HAV vaccine is not included in the national immunization program (NIP) in Mexico. This study addresses an identified evidence gap of the burden of hepatitis A disease, complications, and associated costs in Mexico by analyzing surveillance and healthcare data. Data review included disease morbidity (incidence and hospitalization), mortality, and healthcare resource utilization costs.

**Methods:**

In this observational, retrospective database study, we conducted a systematic screening, extraction, and analysis of outcome data from the national surveillance system in Mexico from January 2000 to December 2019.

**Results:**

During the analysis period (2000–2019), the average incidence rate/year of HAV cases was 14.7 (5.4–21.5) per 100,000 inhabitants. Children 1–9 years of age (YoA) had the highest average incidence rate/year with 47.8 (14.7–74.5). The average hospitalization rate/year due to HAV infection was 5.8% (2.9–9.6%). Although the highest burden of HAV continued to be in children (1–9 YoA), an increase in incidence and hospitalizations (with complications) in older age groups (≥ 10–64 YoA) was observed. The annual average fatality rate was estimated to be 0.44% (0.26–0.83%) of which 28.8% of deaths were concentrated in adults ≥ 65 YoA. The total direct costs of medical attention due to HAV and related complications were estimated at $382 million Mexican pesos.

**Conclusion:**

The overall results suggest an uptrend in HAV infections in adolescents/adults compared to children in Mexico. Therefore, as the overall incidence risk of HAV infection decreases, the mean age of infection increases. This consequently increases the risk of severity and complications in older age groups, thus increasing the demand for healthcare resources. Our findings provide evidence for including the inactivated HAV vaccine in the Mexican NIP.

## Introduction

The hepatitis A virus (HAV), classified as a picornavirus, causes hepatitis A infection. Natural hosts of HAV include only humans who typically acquire the virus through ingestion of contaminated food or water or close contact with an infectious person, and spread through sharing of needles, and household or sexual contacts [1]. Transmission is usually through the fecal-oral route [1]. Typical presentations of hepatitis A include a sudden onset of fever, malaise, nausea, abdominal discomfort, dark urine, anorexia, and jaundice [1]. Symptomatic illness is directly related to age; while children < 6 years of age (YoA) are usually asymptomatic, older children and adults are likely to have symptomatic illness and present with acute hepatic damage and jaundice [1,2]. Severe clinical manifestations of hepatitis A infection, although rare, may occur. HAV infection has been registered to be the cause of acute liver failure (ALF) in 81.4% of cases and is frequent in developing countries, with reports of 3.1 to 26% [3]. ALF is more frequent in adults and patients with underlying chronic hepatopathy compared with children. Fulminant hepatitis is the most severe rare complication, with mortality estimates up to 80% [1,3,4]. The severity and clinical outcome following HAV infection is determined mostly by the age of the infected individual, in other words, HAV infection is associated with more severe disease, a higher risk of fulminant hepatitis and death with progressing age of the individual [1].

It is challenging to distinguish hepatitis A from other types of viral hepatitis only on the basis of clinical attributes. A confirmed diagnosis of hepatitis A requires serologic testing, which detects the presence of immunoglobulin M (IgM) anti-HAV in the acute phase of infection and immunoglobulin G (IgG) anti-HAV in the convalescent phase of the infection. Anti-HAV IgM antibodies indicate recent or current infection and IgG antibodies indicate past infection and usually persist throughout an individual’s lifespan after infection or vaccination [1]. Together, these measures also prove useful in providing important epidemiologic insights and HAV seroprevalence estimates [5]. There are four levels of HAV endemicity as defined by the World Health Organization (WHO) on the basis of seroprevalence (anti-HAV IgG): high (≥ 90% by 10 YoA); intermediate (≥ 50% by 15 YoA, with < 90% by 10 YoA); low (≥ 50% by 30 YoA, with < 50% by 15 YoA); and very low (< 50% by 30 YoA) [6].

Globally, hepatitis A disease occurs sporadically, with a trend of cyclic recurrences [7]. HAV is estimated to be responsible for approximately 100 million clinical cases of viral hepatitis every year of which only about 1.5 million cases are reported [8,9]. This suggests a gross underreporting of HAV infections (~ 80%) primarily due to a substantial number of asymptomatic infections in younger age groups [9]. In high-income countries, hepatitis A accounts for 20–25% of the total viral hepatitis burden whereas this burden is expected to be higher in low- and middle-income countries [10,11]. Over the last two decades, improving socioeconomic indicators such as rising incomes and access to clean water and sanitation [12–14] have led to an evolution in HAV seroepidemiology with several countries transitioning from high to intermediate and intermediate to low endemicity.

Latin America is a region with heterogeneous levels of HAV endemicity, mostly ranging from high-intermediate to low-intermediate [15]. In Mexico, HAV is the leading cause of viral hepatitis in the general population (79.0%) [16], and is the most common cause of a viral infection in children. The seroprevalence of hepatitis A in Mexico has been described in previously published literature [17]. Using the 2012 National Health and Nutrition Survey data, the mean weighted HAV seroprevalence was reported to be 69.3% (95% confidence interval [CI]: 64.8–73.4) overall, with 58.8% (95% CI: 53.4–64.1) in adolescents and 83.0% (95% CI: 75.3–88.7) in young adults. By 10 YoA, 46.7% (95% CI: 33.9–60.0) of children were seropositive and by 15 YoA this rate increased to 52.8% (95% CI: 36.5–68.5), thus corresponding to an intermediate endemicity nationwide [15,17,18]. However, this seroprevalence data does not describe the actual burden of symptomatic disease due to HAV infection or the disease complications among children and adults [19], which could help inform the development of appropriate disease management strategies such as vaccination.

WHO recommends that vaccination against HAV be integrated into the national immunization schedule for children ≥ 1 year of age if indicated on the basis of incidence of acute hepatitis A, change in the endemicity from high to intermediate, and consideration of cost-effectiveness [6]. Despite the recommendation by WHO, the pediatric HAV vaccine is not included in Mexico’s national immunization program (NIP) [6,17,18]. Although since 2013, a single dose of hepatitis A vaccine is recommended for infants of agricultural workers and infants living in shelters and nurseries (children at Instituto Mexicano del Seguro Social [IMSS] daycare centers). A similar recommendation was made for children in the age group of 1 to 5 years living in shelters undergoing an acute diarrheal disease outbreak [20]. However, these recommendations cover only a minority of the total Mexican pediatric population. It should be noted that despite these recommendations, there is no information on the coverage, impact, and cost-effectiveness of this vaccine at national or regional level.

**Fig 1:**
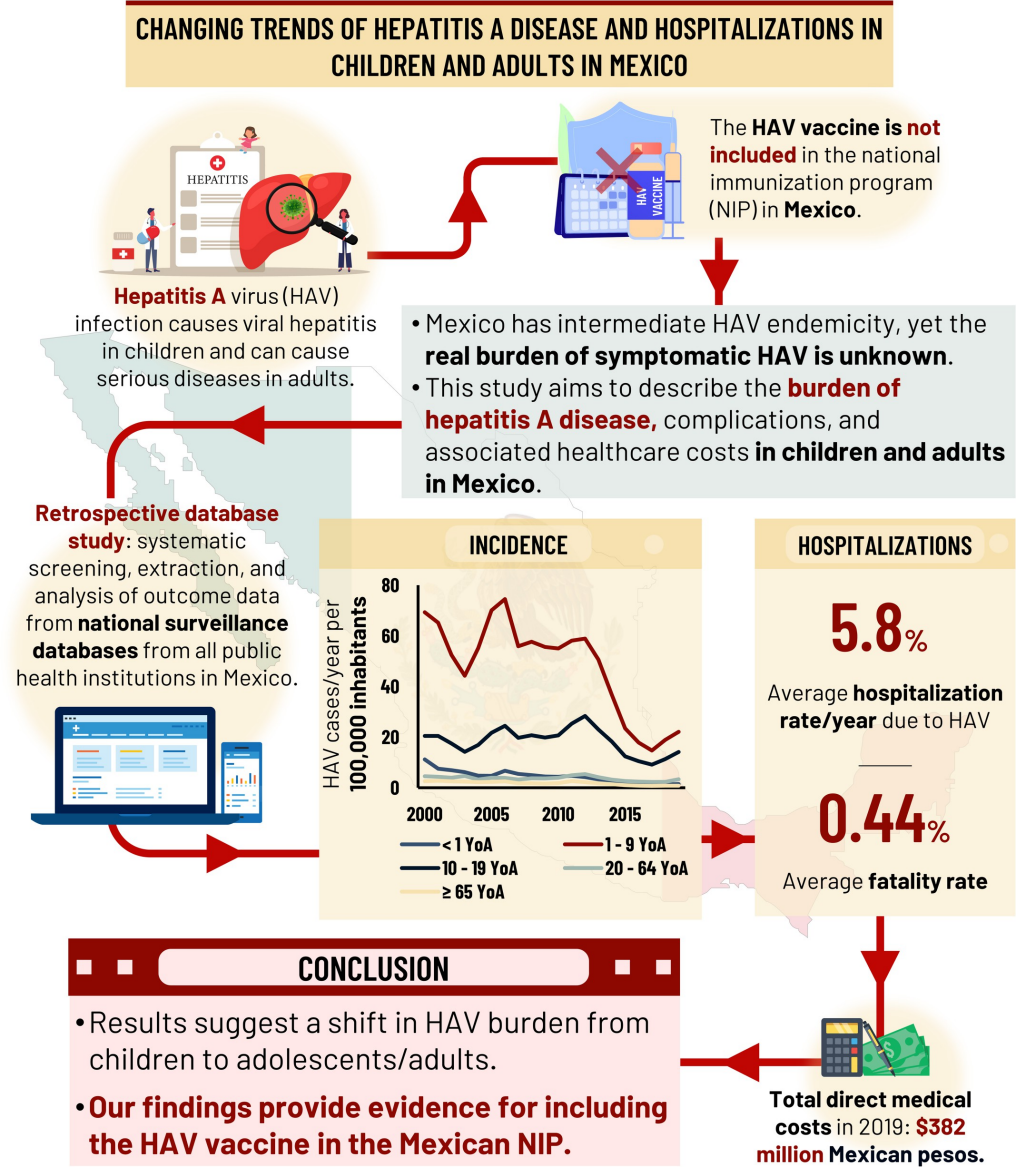
Graphical abstract.

This study aims to fill the evidence gap in published literature regarding the actual burden of symptomatic disease due to HAV infection or its complications in children and adults in Mexico [17,19]. The main objective of this study was to describe the burden of hepatitis A and associated complications such as icteric syndrome, fulminant hepatic failure (FHF), ALF, and liver transplant (LT) due to HAV infection in children and adults in Mexico from January 2000 to December 2019. Other objectives of this study were to describe the burden of disease due to HAV infection in regions or states in Mexico with low-medium endemicity and high infant mortality; and to estimate healthcare resource utilization due to HAV and its complications in children and adults in Mexico. For a short summary of this article and its findings, please refer to the graphical abstract accompanied with this manuscript **(Fig 1)**.

## Materials and methods

### Study design and population

A retrospective database study was performed using national surveillance data covering all public health institutions in Mexico. Data on laboratory or clinically confirmed HAV cases, hospitalizations, and deaths, including those related to severe complications of hepatitis A were analyzed from January 2000 to December 2019. The target population was divided into children (< 1, 1–4, 5–9, and 10–14 YoA), adolescents (15–19 YoA), young adults (20–24 YoA), adults (25–44, 45–49, and 50–59 YoA), and older adults (60–64 and ≥ 65 YoA) in Mexico. Individuals who reported with HAV infection (cases, hospitalizations, deaths), any icteric syndrome, FHF, ALF, and LT due to HAV infection (cases, hospitalizations, deaths) in the national databases of Mexico, were included. Sample size estimation was not applicable, since this study used all the available aggregate data.

Due to the observational study design, there was no prospective data collection involved and all data was obtained from the publicly available Mexican national surveillance database, which is comprehensive of the whole population. As such, an ethics approval from the regulatory and bioethics committees did not apply. Further details can be found in the appendix of the study protocol (available upon request at www.clinicalstudydatarequest.com).

### Case definitions

This study included all available aggregated data of confirmed cases, hospitalizations, and deaths due to HAV infection and its related complications. Case definitions of HAV infection and associated complications were identified by utilizing the International Statistical Classification of Diseases and Related Health Problems, Tenth Revision (ICD-10) (Table 1) [21]. When defining the disease complications, the primary diagnoses were first identified and were then correlated with the secondary and specific diagnoses of HAV infection.

**Table 1:** Definition HAV-associated disease based on ICD-10 codes [21].

Disease outcome		ICD-10 code
Confirmed cases and deaths due to HAV	Acute hepatitis A	B15
	Hepatitis A with hepatic coma	B15.0
	Hepatitis A without hepatic coma	B15.9
Complications due to HAV infection^a^	Icteric syndrome	R17
	(Fulminant) Hepatic failure	K72.1, K72.9
	Acute liver failure	K72.0
	Liver transplant	T86.4

HAV: Hepatitis A virus; ICD-10: International Statistical Classification of Diseases and Related Health Problems, Tenth Revision.

^a^ Served as primary diagnosis when identifying a case of complication, which is subsequently correlated with the diagnoses specific to HAV infection.

### Data sources

Based on the case definitions, relevant data for this study was collected from various databases [22–33], which encompass the complete public national surveillance system in Mexico, thus we assumed that the data had 100% national coverage (**S1 Table in S1 File**). Total number of cases (suspected or confirmed) due to HAV infection were obtained from Anuarios de Morbilidad 1984–2018/Dirección General de Epidemiologia (DGE) [23]; Boletín Epidemiológico/Sistema Nacional de Vigilancia Epidemiológica/Sistema Único de Información/Dirección General Adjunta de Epidemiologia (DGAE) [24], which are consolidated in the information platform “Sistema Nacional de Vigilancia Epidemiológica (SINAVE)”; and Statistical Yearbooks of Public Healthcare Institutions [25–27]. Data on hospitalizations and deaths due to HAV infection and its related complications were obtained from Cubos Dinámicos/Dirección General de Información en Salud (DGIS) [28]. To complete the missing information or data not updated to 2019 (i.e., cases, hospitalizations, and deaths due to HAV infection and its complications), we obtained data through the Instituto Nacional de Transparencia, Acceso a la Información y Protección de Datos Personales (INAI) [29] and Centro Nacional de Trasplantes (CENATRA) [30]. Population estimates relevant to the study population were obtained from the Consejo Nacional de Población (CONAPO) database [22].

Direct medical costs related to HAV infection and its complications were estimated from a Delphi expert panel survey: the Costos Unitarios por Nivel de Atención Médica from the Instituto Mexicano de Seguridad Social (IMSS) [31]; Grupos Relacionados con el Diagnóstico (GRD’s) from the IMSS [32]; and COMPRANET 5.0 [33].

### Study outcomes

Outcomes related to the description of burden of disease due to HAV infection among children and adults in Mexico included case count and incidence proportion, hospitalization count and proportion, and death count and mortality. The same outcomes were selected for the description of complications due to HAV infection (icteric syndrome, FHF, ALF, LT) and HAV infection in states with low-medium endemicity and high infant mortality. To estimate the direct medical costs of HAV infection in children and adults, outcomes included resource use, inpatient and outpatient cost related to laboratory and cabinet studies, hospitalization day bed, surgical medical procedures, and medicines, among others, for the management of patients with HAV infection and each of the potential complications.

### Analyses

The descriptive statistics for all the variables related to case, hospitalization, and death occurrence and complications due to HAV infection were provided in terms of number (n), frequency, and incidence/mortality (number of events per population). The descriptive statistics were also provided by month and year or only year (as applicable), by age, gender, and region. For the outcomes related to the burden of disease in states with low-medium endemicity and high infant mortality, an analysis was conducted by stratifying the primary objective outcomes into low-medium endemicity and high infant mortality states/regions; and if possible, compared with the regions of high endemicity. Total population by age groups from the CONAPO database [22] was used to calculate the outcome measures in age groups of interest.

To estimate healthcare resource utilization a data collection survey was disseminated by e-mail to collect information from an expert Delphi panel, which consisted of 16 specialists in pediatrics, infectiology, internal medicine, gastroenterology, hepatology, and surgery from the public sector of healthcare attention in Mexico. A first round was carried out in which the data recollection tool was sent to the panelists via e-mail. The responses were captured and analyzed to obtain the minimum, median, and maximum points. A second round was carried out where the results of the first round were sent via e-mail and where the panelists were invited to give their arguments if they were at the extreme points (minimum or maximum), aiming for a consensus through the analysis of each point. Afterwards, the panelists were invited to answer a second questionnaire. This allowed us to obtain resource consumption rates linked to medical care of patients with HAV infection and its complications. We also used local sources to estimate unit costs for medical care of attention [31] and treatment costs [33]. The respective costs were then applied to each item to estimate the cost per capita and the total cost of medical care of attention at national level. Medical healthcare costs of attention for 2019 based upon four health conditions related to HAV infection: asymptomatic non-icteric HAV infection; symptomatic (icteric) HAV infection; HAV infection + FHF/ALF; and HAV infection + FHF/ALF + LT. Cost data are presented in Mexican peso ($, MXN).

For all outcomes, a descriptive statistical analysis was conducted to estimate the total number of occurrences, mean, median, 95% CI, and p-value (for differences by year) for the different outcomes. Results are presented as overall and by groups stratified by gender, age, region, or state (North, South, Center, Mexico City [CDMX]), and year.

## Results

### Population characteristics

During the study analysis period (2000–2019), the total population of Mexico increased by 28% from 98,785,275 inhabitants in year 2000 to 126,577,691 inhabitants in year 2019. Gender distribution remained constant over the same period, corresponding to 51.0% females and 49.0% males in the total population.

During the analysis period (2000–2019), there were a total of 324,022 cases, 16,996 hospitalizations and 1,434 deaths due to HAV at a national level in Mexico among all age groups **(Table 2)**.

**Table 2:** Total numbers of cases, hospitalizations and deaths due to HAV in Mexico from 2000–2019 by age group.

Age group	Total number of cases	Total number of hospitalizations	Total number of deaths
Total < 1 YOA	2,018	507	43
Total 1–4 YOA	68,744	2,726	287
Total 5–9 YOA	124,865	3,687	97
Total 10–14 YOA	58,492	2,913	68
Total 15–19 YOA	22,617	1,993	44
Total 20–24 YOA	13,961	1,457	39
Total 25–29 YOA	11,565	1,035	47
Total 30–34 YOA	6,440	626	40
Total 35–39 YOA	2,070	439	49
Total 40–44 YOA	2,866	327	50
Total 45–49 YOA	2,977	260	52
Total 50–54 YOA	2,526	243	62
Total 55–59 YOA	1,257	195	68
Total 60–64 YOA	1,231	170	75
Total ≥ 65 YOA	2,393	418	413
**Totals**	**324,022**	**16,996**	**1,434**

HAV, hepatitis A virus; YoA, years of age.

### HAV incidence

**Fig 2:**
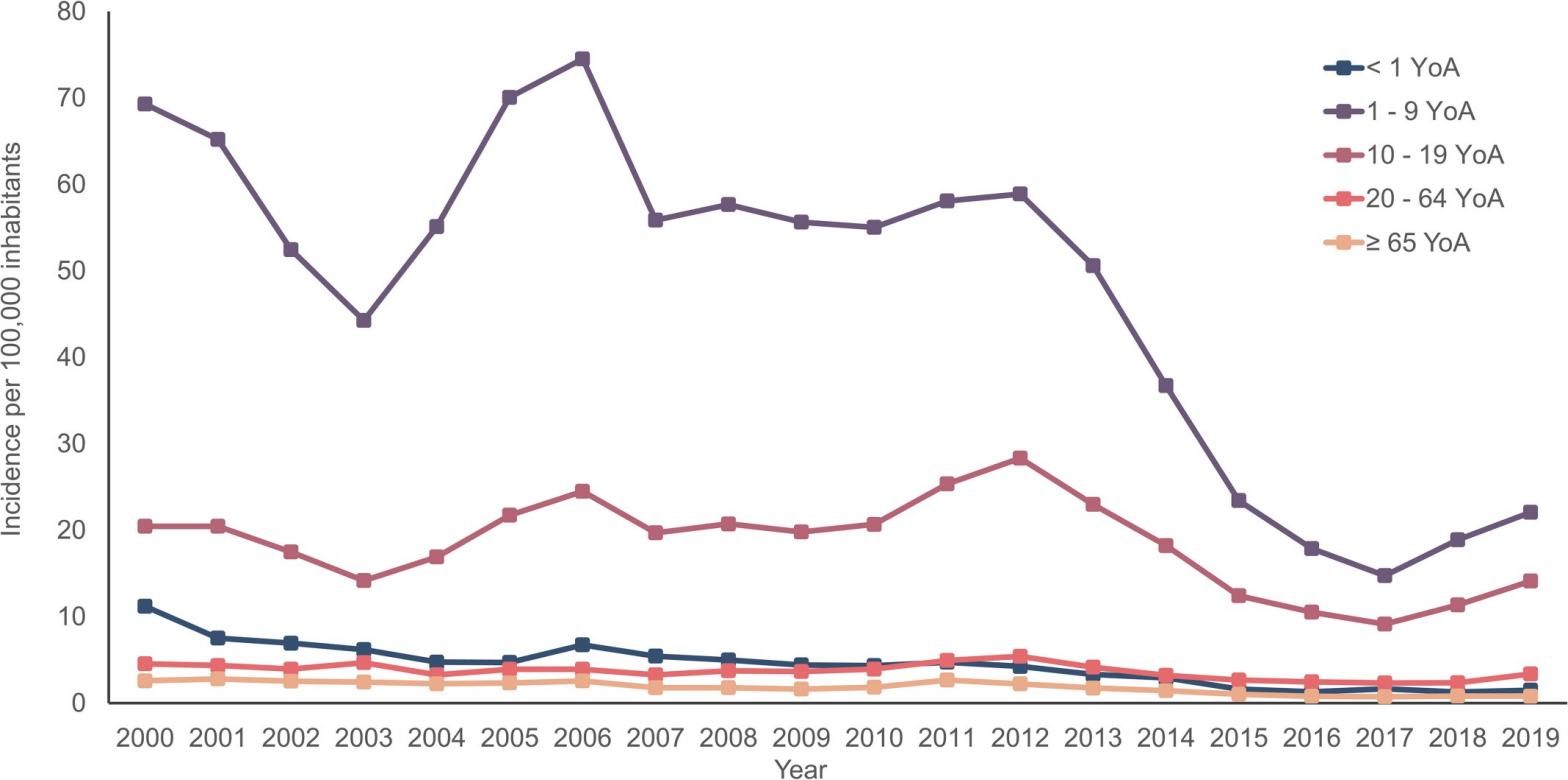
Incidence rate for diagnosed/detected HAV cases by age group and year. YoA: Years of age.

During the analysis period (2000–2019), the annual average incidence rate of HAV cases was 14.7 (5.4–21.5) per 100,000 inhabitants. Two age groups: 1–9 YoA (47.8; range: 14.7–74.5) and 10–19 YoA (18.5; range: 9.2–28.3) had the highest incidence rates of HAV cases per 100,000 inhabitants during the analysis period (**Fig 2**). A noticeable predominance of HAV cases in adult males was observed with an annual average incidence rate of 15.7 (range: 5.9–22.9) per 100,000 inhabitants in comparison with the female population (**S1 Fig in S1 File**). Regionally, in year 2000, the CDMX region had the highest incidence rate (26.8 per 100,000 inhabitants) compared to the other regions. However, in year 2010 the Center (19.4 per 100,000 inhabitants) and the CDMX (18.6 per 100,000 inhabitants) regions presented the highest incidence rates (**S2 Fig in S1 File**). In year 2019, there was a significant reduction in the incidence of HAV cases nationwide, but incidence in the South region of the country was twice the amount of the CDMX region (10.1 per 100,000 inhabitants and 5.1 per 100,000 inhabitants, respectively). Overall, we observed a homogeneous decrease in HAV incidence with a recent increase in the national incidence, particularly in the South region since the year 2017 (**S2 Fig in S1 File**).

### Hospitalizations due to HAV

**Fig 3:**
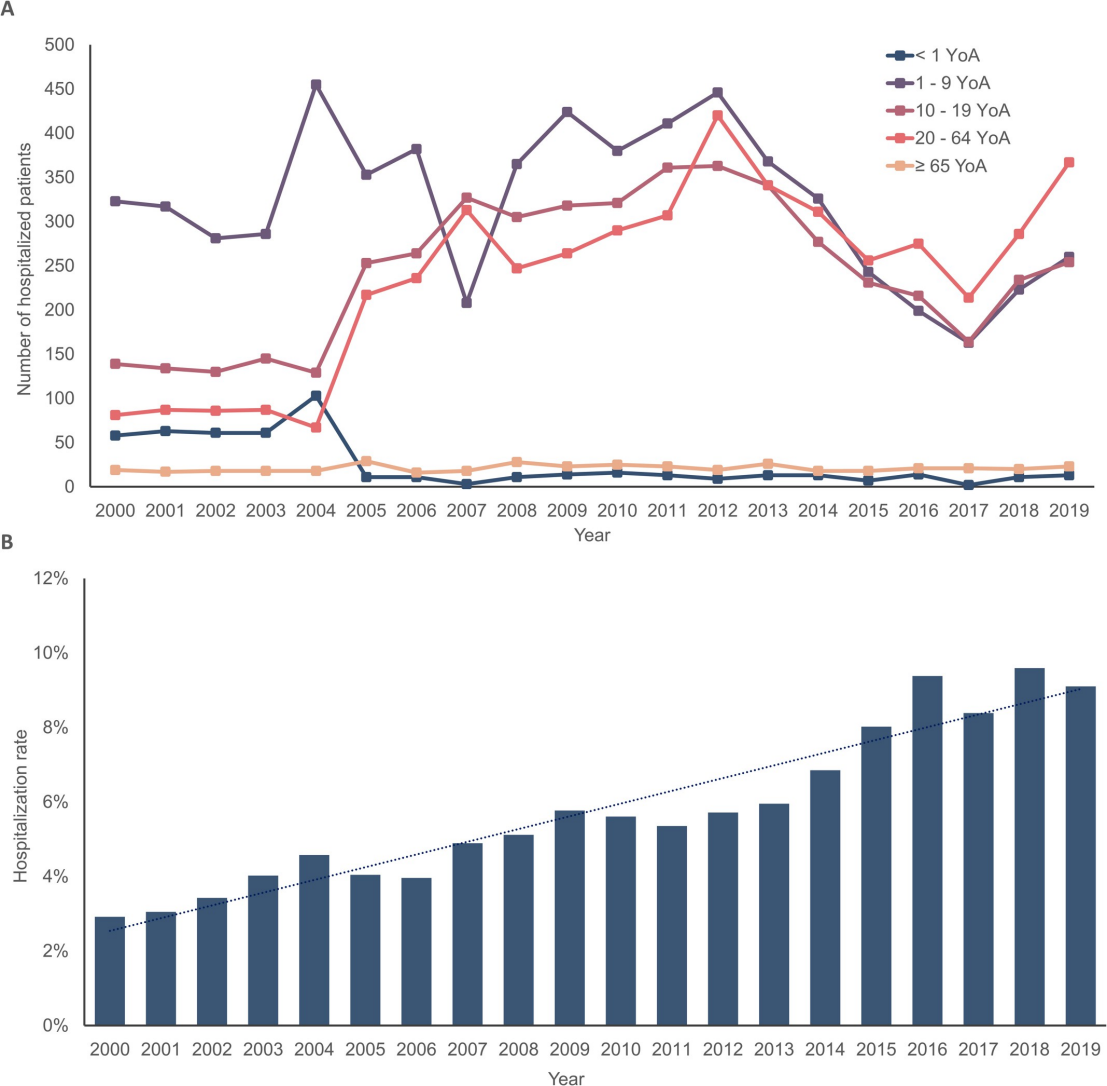
Total number of hospitalizations and hospitalization rates for HAV infection by age groups in Mexico throughout the observation period (2000–2019). A) Total sum of patients hospitalized for HAV infection by age group in Mexico throughout the observation period (2000–2019). B) Hospitalization rate due to HAV infection by year in Mexico throughout the observation period (2000–2019). HAV, hepatitis A virus; YoA, years of age.

On average, 850 patients (range: 564–1,257 patients) were hospitalized due to HAV infection per year. No differences were observed between females and males among patients hospitalized due to HAV infection. We observed that children from 5–9 YoA had the highest number of hospitalized patients (184 average patients/year, range: 76–309 patients), followed by 10–14 YoA (146 average patients/year, range: 90–206 patients), and 1–4 YoA (136 average patients/year, range: 64–335 patients). Considering cases from the 20–64 YoA group, we observed an annual average of 238 patients (range: 81–420). Individuals least affected with hospitalization due to HAV infection were < 1 and ≥ 65 YoA (annual average/cases: 25 and 21, respectively) (**Fig 3A**). Regionally, the states with ≥ 5.0% of patients hospitalized due to HAV infection from years 2000 to 2019 corresponded to CDMX, South (Chiapas, Oaxaca, Veracruz), and the Center (Guanajuato, Jalisco) regions (**S3 Fig in S1 File**).

The average hospitalization rate/year due to HAV infection from years 2000 to 2019 was 5.8% (range: 2.9–9.6%). From 2000 to 2019, there was an overall increasing trend in the hospitalization rates due to HAV, with two prominent peaks in the years 2016 and 2018 that correspond to the increase in hospitalizations in the 20–64 and 10–19 YoA groups (**Fig 3B, S2 Table in S1 File**).

### Hospitalizations due to HAV-related complications (FHF or ALF)

An average of 19 patients (range: 9–26 patients) per year were hospitalized due to HAV-related complications such as FHF or ALF. Hospitalizations due to HAV with FHF or ALF represented an annual average of 2.3% (range: 1.0–4.3%) during the observation period (2000–2019).

**Fig 4:**
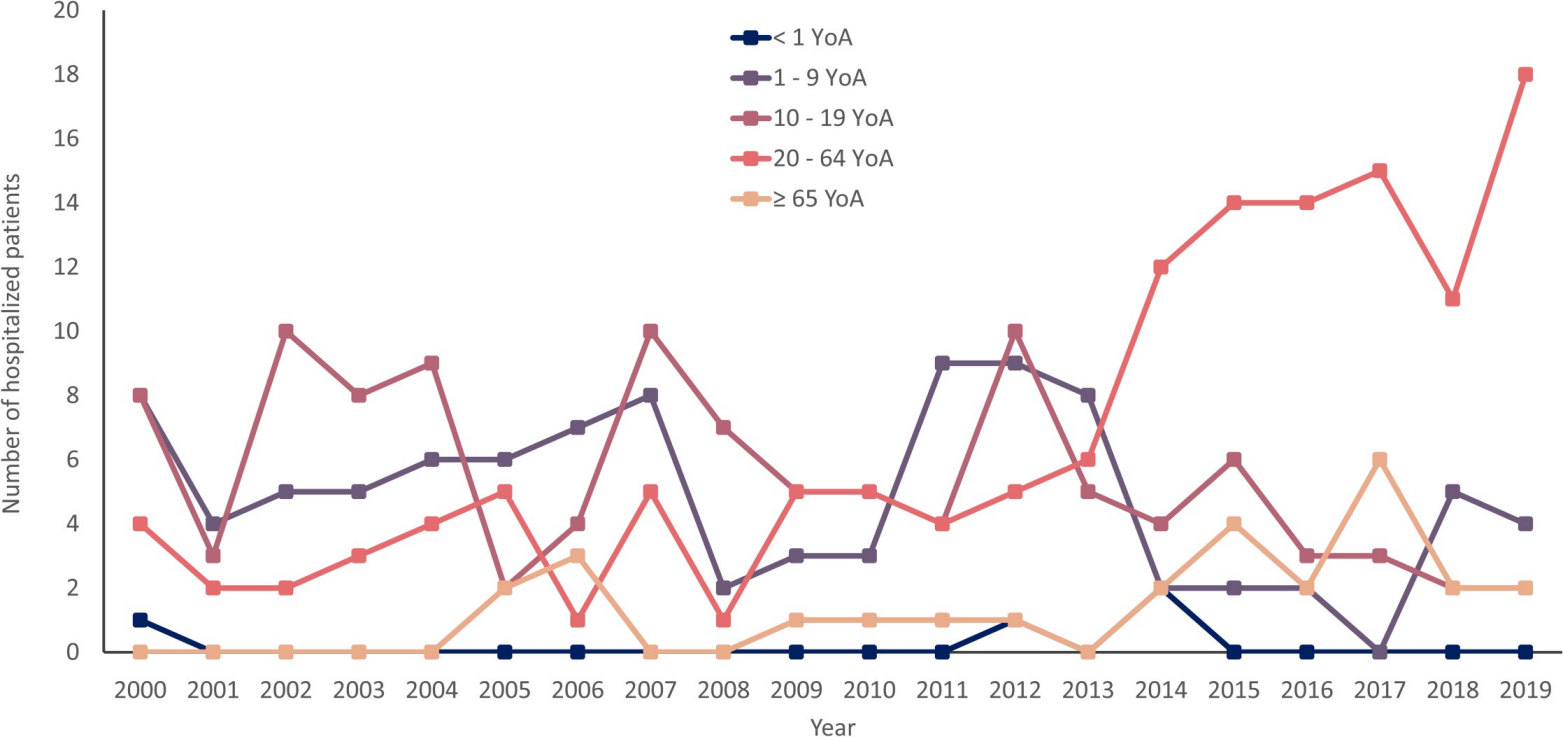
Hospitalizations due to HAV +FHF or ALF infection by age group and year in Mexico. YoA, years of age.

The gender of patients hospitalized due to HAV + FHF or ALF infection, was 54.9% for males (range: 41.2–71.4%) and 45.1% for females (range: 28.6–58.8%). During the complete observation period (2000–2019), we observed that the age group with the highest number of hospitalized patients due to HAV + FHF or ALF corresponded to children 10–14 YoA (annual average of four patients, range: 0–7 patients), followed by age groups 5–9 YoA (annual average of three patients, range: 0–7 patients). The age group with the least burden corresponded to children < 1 YoA. However, since the year 2014 to 2019 there was an increasing trend in patients between 20–64 YoA that were hospitalized due to HAV + FHF or ALF infection (**Fig 4**). In year 2000, regions with ≥ 5.0% of patients hospitalized for HAV + FHF or ALF were CDMX, Guanajuato, Jalisco, and Puebla (which represented 9.5% each), and Sonora (14.3%). In year 2010, regions with ≥ 5.0% of patients hospitalized for HAV + FHF or ALF were Chihuahua, Guanajuato, State of Mexico, Morelos, Oaxaca, Queretaro, Sinaloa, Sonora, and Tabasco where each state represented 7.1% of the cases while Jalisco and CDMX represented 21.4 and 14.3%, respectively. Finally, in year 2019 regions with ≥ 5.0% of patients hospitalized for HAV + FHF or ALF were Chiapas, Jalisco, and Tabasco, which represented 7.7% of the hospitalized cases each, CDMX and Sinaloa 11.5% each, and the State of Mexico (15.4%) (**S4 Fig in S1 File**).

### LT due to FHF or ALF

**Fig 5:**
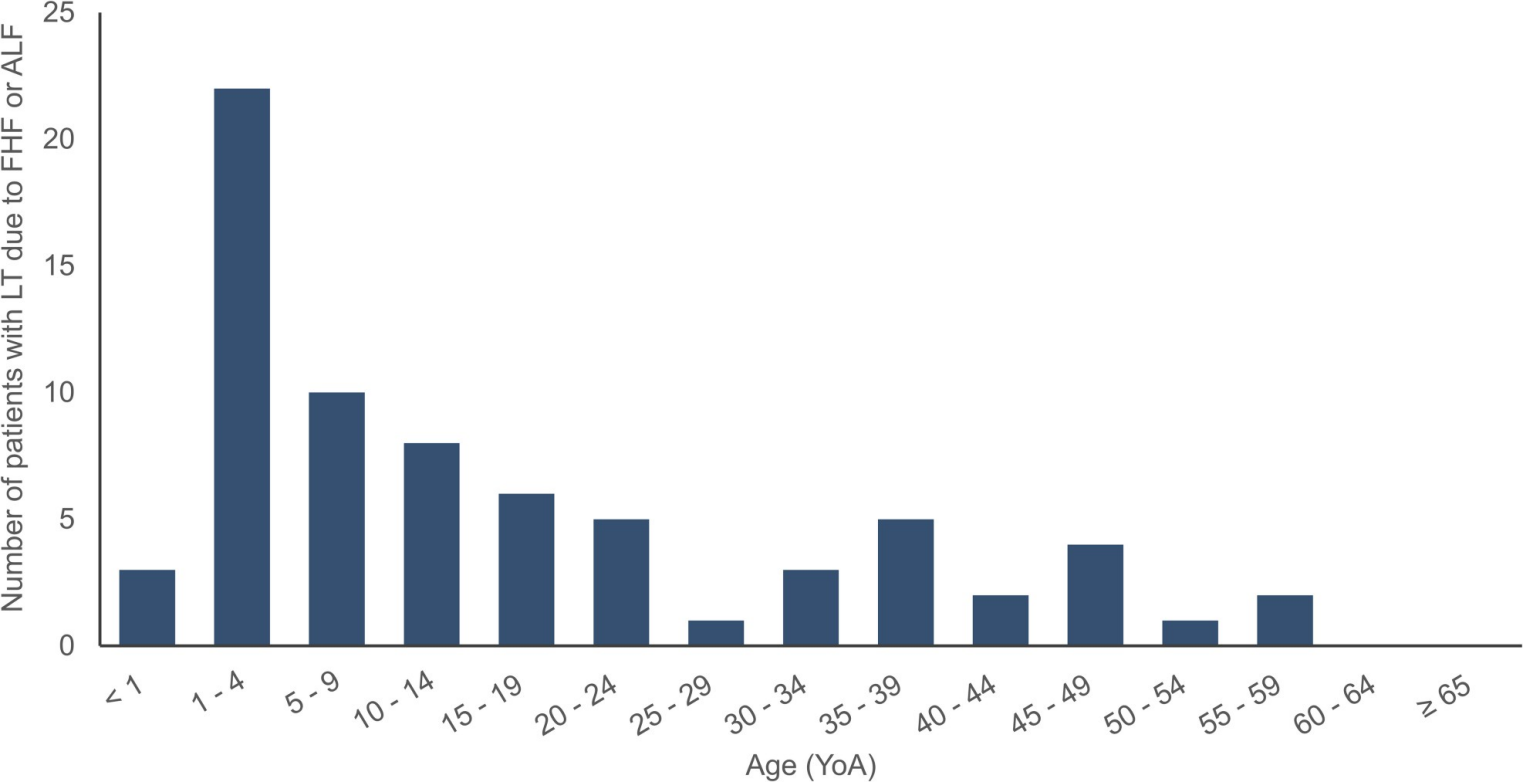
Total sum of patients with LT due to FHF or ALF by age group in Mexico throughout the observation period (2007–2019). ALF: Acute liver failure; FHF: Fulminant hepatic failure; LT: Liver transplant; YoA: Years of age.

According to the CENATRA, LT registration began in Mexico from year 2007. In year 2019, about 1,334 LT due to any cause had been performed. On average 5.4% (range: 1.8–9.4%) of all LT were due to FHF/ALF. We observed 72 accumulated cases of LT due to FHF/ALF with 80.6% of the cases among < 35 YoA. No transplants were performed in patients ≥ 60 YoA and the patients ≥ 35 YoA represented 19.4% of all LT due to FHF/ALF (Fig 5). Females represented 68.1% of patients with LT due to FHF/ALF. Regionally, cases of LT due to FHF/ALF were mainly concentrated in the CDMX region with 54.2% of the cases, followed by Nuevo Leon (27.8%), Jalisco (12.5%), San Luis Potosi (2.8%), and Sinaloa (2.8%) (S5 Fig **in S1 File**). No data was found or reported for patients with LT due to FHF/ALF secondary to HAV infection in the databases. Thus, we estimated that approximately 9.0% of LTs due to FHF/ALF secondary to a HAV infection were performed in Mexico, as reported in a previous publication [34].

### Deaths reported due to HAV Infection

**Fig 6:**
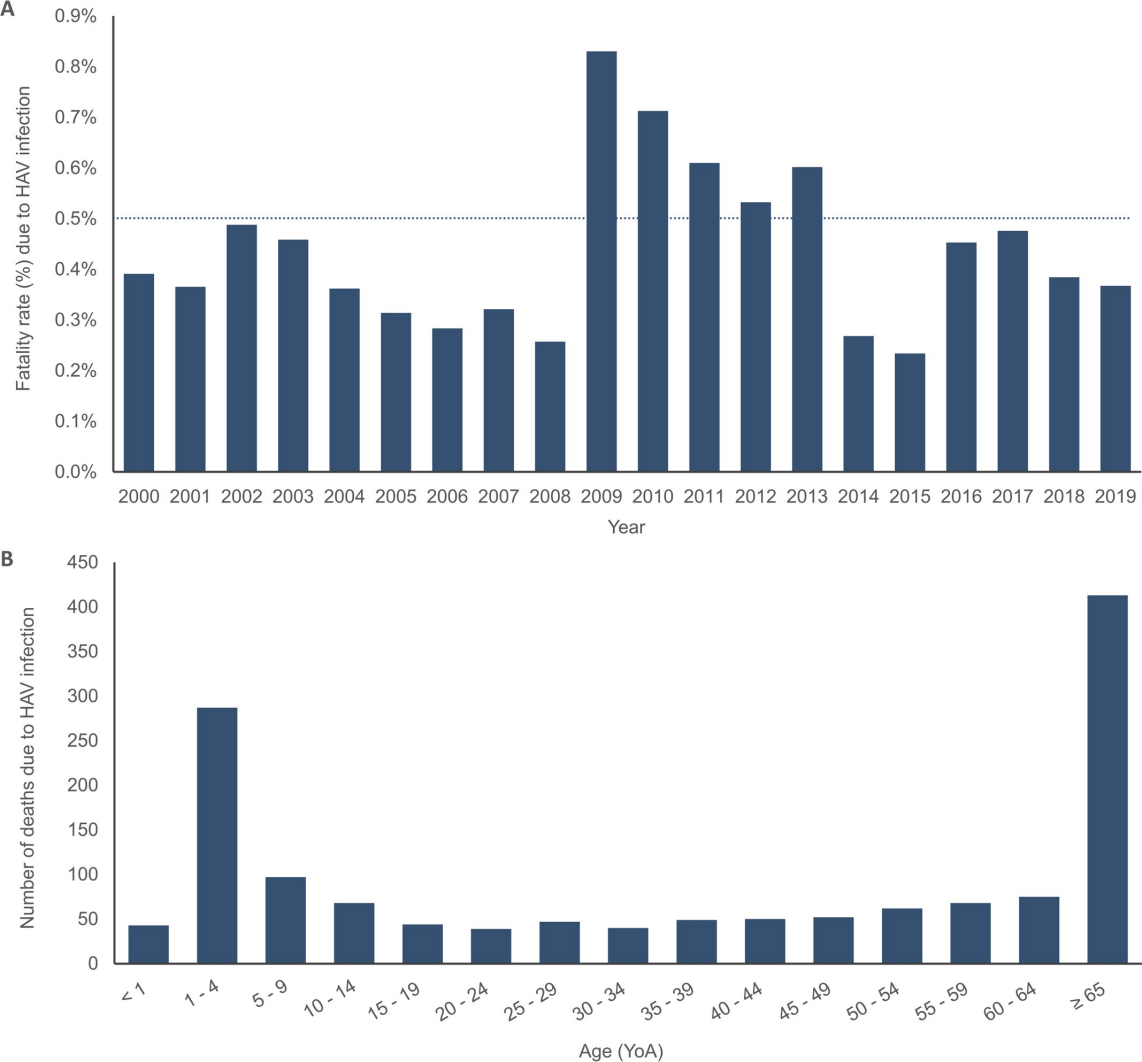
Deaths due to HAV infection by year and age group in Mexico throughout the observation period (2000–2019). A) Fatality rate (%) due to HAV infection by year in Mexico throughout the observation period (2000–2019). B) Total sum of deaths due to HAV infection by age group in Mexico throughout the observation period (2000–2019). HAV, hepatitis A virus; YoA, years of age.

From 2000 to 2019, out of a total of 1,434 deaths due to HAV registered at the national level, the annual average number of deaths was 72 (range: 22–150). Based on the total number of deaths per year the annual average fatality rate was estimated to be 0.44% (0.26–0.83%) during the observation period (Fig 6A). Approximately 28.8% of deaths were reported in adults ≥ 65 YoA, followed by 20.0% in children 1–4 YoA (Fig 6B). Fatality rate due to HAV sharply increased in 2009 for the following 5 years, and then after 2013 decreased greatly. The majority of deaths registered during the observation period were among females with 54.8%. The regions with ≥ 5.0% of deaths registered due to HAV infection corresponded to CDMX (11.9%), State of Mexico (8.2%), Puebla (7.9%), Veracruz (7.8%), Chiapas (6.4%), Jalisco and Oaxaca (6.3%, respectively), and Guanajuato (5.4%) (S6 Fig **in S1 File**).

### Direct medical costs and resource utilization related to HAV cases

Asymptomatic non-icteric HAV infection. For year 2019, we estimated a total of 1,022 (10.1%) patients with “asymptomatic non-icteric” disease. It was estimated that the cost of medical care for these patients ranged between $11,810.1 MXN and $13,573.4 MXN. These costs included outpatient management, laboratory, and cabinet studies, as well as follow-up visits. The total cost of care for the patient cohort was $12,519,306.6 MXN (S3 Table **in S1 File**).

Symptomatic (icteric) HAV infection. The cases of HAV infection with symptomatic disease (icteric) represented 89.6% (9,026) of the total cases treated in year 2019. The average cost of medical care of attention ranged between $40,089.0 MXN and $45,389.4 MXN among different age groups. The costs generated by hospitalization including emergency room attention, hospitalization/day and intensive care unit/day, laboratory, imaging and radio diagnostic studies, and pharmacologic treatment, as well as outpatient management (including medical visits, laboratory, imaging and radio diagnostic studies, and pharmacologic treatment) were considered. The total cost of care for the patient cohort was approximately $365.4 million MXN (S4 Table **in S1 File**).

HAV infection + FHF or ALF. During year 2019, 26 patients were diagnosed with HAV infection + FHF/ALF, which represented 0.26% of the total number of patients diagnosed for HAV infection. The average cost of medical care of attention for these patients ranged from $68,604.5 to $154,883.6 MXN. The age group most affected by this health condition corresponded to adults 20–64 YoA (69.8%), which represented 74.1% of the total cost generated by this health condition (S5 Table **in S1 File**).

HAV infection + FHF or ALF + LT. For year 2019, from a total of 13 patients who underwent LT due to FHF/ALF, one patient presented as secondary to HAV infection. The cost for year 2019 was estimated to be $1,262,232.6 MXN (S6 Table **in S1 File**), based on the LT costs as published by the IMSS GRD’s for year 2018.

Total costs of medical attention for all patients with HAV infection in year 2019. The estimated total cost of medical care of attention in year 2019 for 10,075 patients due to HAV infection and its related complications was estimated to be $382,862,577.3 MXN (Table 3). Age groups with the highest cost burden were children 1–9 YoA and young adolescents 10–19 YoA. Both groups cumulatively represented 74.5% of the total cases and 73.5% of the total costs. The age group 20–64 YoA alone represented ~ 25.0% of both inpatient and total costs (Table 3). The total direct costs for medical care of attention due to HAV and related complications (in year 2019) was estimated at $382,862,577.3 MXN.

**Table 3:** Total cost of medical care of attention for patients with HAV infection and related complications (year 2019).

Age group	Total patients with HAV infection and related complications (year 2019)	Total medical costs ($ MXN, year 2019)
< 1	32	1,351,722.2
1–9	4,372	162,992,758.5
10–19	3,136	119,507,325.1
20–64	2,459	95,803,168.9
≥ 65	76	3,207,602.6
Total	10,075	382,862,577.3

HAV: Hepatitis A virus; $ MXN: Mexican peso.

## Discussion

This study fills a gap in the public health, medical, and epidemiologic knowledge regarding the burden of hepatitis A disease, complications, and associated costs in Mexico. We analyzed national surveillance and healthcare data in Mexico to show the magnitude of the disease related to symptomatic or severe HAV infection, which complements previous seroprevalence studies in Mexico [17].

### Primary objectives

Overall, there is evidence of a decreasing trend in HAV disease among children, however, there is an increasing trend in HAV disease and complications in older age groups, which is expected in countries with intermediate endemicity like Mexico. The implications of HAV at older ages mean risk for more severe and complicated disease, and greater demand on healthcare resources since increasing age is a risk factor for HAV. There were also notable changes in trends of hospitalization and deaths over time and across ages, with symptomatic cases and hospitalizations increasing with time in older age groups. Although there is a substantial decrease in overall trends of HAV disease from years 2000 to 2019 (especially after year 2013), we can also observe a slightly increasing trend in hospitalizations and incidence from year 2017 until year 2019. As the risk of hepatitis A infection declines, the mean age of infection increases (i.e., intermediate endemicity), where the risk of symptomatic disease is higher. The shift in the mean age at infection, from younger age groups to older age groups, increases the population-level burden of disease. This occurs due to the increased severity and associated complications of the disease among older age groups. A previous HAV transmission modeling study conducted in Brazil and Mexico provides similar evidence [35]. Over time, the hospitalizations and deaths increase among individuals of older ages. Specifically, as hospitalization rates increase over time and incidence decreases, it could be interpreted as an increase in disease severity. Additionally, after day-care HAV vaccination was implemented in 2013, there was a sharp decline in incidence, which translated to a proportional decrease in fatality rates particularly in the pediatric population. This fact provides a strong rationale to implement HAV vaccine in the Mexican NIP without further delay.

### Secondary objectives

Significant variation is observed in the incidence of HAV across regions although consistent with the overall age-specific trend of a shift in endemicity. Regionally in year 2000, the highly industrialized CDMX region had the highest HAV incidence (26.8 per 100,000 inhabitants) compared to the other regions. In lower socioeconomic regions, the number of cases could have been underreported due to the incidence of asymptomatic cases and deficient healthcare services. However, for year 2010, the Center (19.4 per 100,000 inhabitants) and the CDMX (18.6 per 100,000 inhabitants) regions presented with the highest incidence. In year 2019, there were lower incidences of HAV cases overall, compared to the past 10 years, but the South region of the country doubled the CDMX region in terms of incidence (10.1 per 100,000 inhabitants and 5.1 per 100,000 inhabitants, respectively). Notably, after 2017 there is an increasing trend in overall National HAV incidence with the South region having the highest incidence by 2019. Mexico is a region with heterogeneous levels of intermediate HAV endemicity, mostly ranging from high-intermediate to low-intermediate, supporting the introduction of disease prevention strategies, as recommended by WHO [6].

A notable finding of this study relates to the pattern between HAV disease, the risks of complications, and the sex of the patient. Further investigation is also required to understand and interpret the reasons for most transplants due to FHF/ALF occurring in the 1–4 YoA group, considering that the highest hospitalization rate due to HAV+FHF/ALF is observed in the 5–14 YoA group. Perhaps these transplants are not HAV-related or were required due to comorbidities or perhaps age is one of the parameters considered for transplants. Also, it may be easier for patients in the 1–4 YoA group to find a transplant organ considering their height and weight.

We found that HAV infection and its complications levy a considerable amount of healthcare resource utilization costs on society and the national healthcare services of Mexico. In terms of costs, our finding suggests that the total direct costs for medical attention due to HAV and related complications (in year 2019) was estimated to be $382,862,577.3 MXN (about $19 million US dollars). When this cost is compared to the total expenditure for year 2019 on the federal public budget for healthcare ($606,602,340,000.0 MXN) [36], the total direct costs represent about 0.063%.

HAV vaccination strategies may vary, depending on the country and HAV endemicity, the risk groups involved, the possibility of post-exposure protection and the cost of vaccination. Several countries have implemented NIP strategies against HAV, and studies demonstrate the impact of both one and two dose vaccination strategies in Latin America and globally, showing similar patterns of incidence reduction [37]. Israel was the first country to implement childhood universal mass vaccination against HAV, vaccinating children 18 months of age. Vaccination coverage reached 90% and 85% for the first and second dose respectively, and as a result the annual incidence of HAV declined within 2–3 years of program initiation, reaching an overall decline of 95% compared with pre-vaccination rates [38–39].

Argentina (one-dose NIP), Panama, Uruguay, and the United States (two-dose NIPs) have reported HAV incidence reductions ranging from 76–96% following introduction of vaccination in the NIP [37]. Argentina reported an 88% reduction (P<0.001), from an average annual rate of 66.5 cases per 100,000 pre-vaccination (2000–2002) to 7.9 cases per 100,000 in 2011 [40]. Panama reported a mean reduction of 93%; baseline case incidence of 51.1/100,000 pre-vaccination (2000–2006) reduced to 3.7/100,000 in 2010 [41]. Uruguay reported a 96% reduction, from 69.6 cases per 100,000 pre-vaccination (2005) to 2.7 cases per 100,000 in 2010 (P<0.05) [42].

### Limitations

Several limitations exist in interpreting the results of this study.

First, as this was an observational retrospective study using data obtained from passive surveillance systems, we could not retrieve disease information for some parts of Mexico where data might not be available.

Second, the presence of various biases such as underreporting/underestimation, discrepancies in case definitions (we used ICD-10 codes [21]), and secular trends, or disease outbreaks might amplify disease burden at specific periods. Underreporting is assumed to be the largest bias, and WHO estimates underreporting of 80% or more cases due to the asymptomatic nature of the HAV infection in children, and shifting endemicity settings [9]. An analysis of underreporting and outbreaks might confound the data; thus, we did not attempt an estimation of the underreported cases in this study.

Third, another limitation addressing the objectives of this study was on the potential multi-morbidity. In older demographics it is to be expected that a person might suffer from more than one of the underlying conditions under study and hence, the incidence for a specific condition might be over- or underestimated based on the comorbidities. It also may point to the more severe outcomes of HAV infections in patients with comorbid conditions and or coinfections.

Fourth, linking diagnostic data with healthcare resource utilization based on the available secondary data was not possible or was only possible to an extremely limited extent. Thus, only hospital stays and prescription of certain single indication drugs (without off-label use) could be causally associated with a certain disease.

Finally, this study did not address the indirect costs to the individual and the society, which are essential for a more accurate and realistic estimation of the total costs of HAV infection and its complications.

### Conclusions

The findings from this study indicate an increase of HAV infection from children to adolescents and adults, which increases the risk for more severe and complicated disease and greater demand on healthcare resources. This study addressed the existing lack of evidence on the burden of HAV infection across all age groups in Mexico.

WHO recommends mass vaccination against HAV in regions, where justified, based on high incidence, declining endemicity, and cost-effectiveness [9]. Similarly, our findings strongly support the inclusion of HAV vaccine in the NIP in Mexico to reduce HAV incidence rates and associated healthcare burden.

## Supporting information

S1 File The supporting information file containing multiple supporting figures and tables, specifically. S1 Table. Overview of study sources. S2 Table. Hospitalized rate (%) comparison for HAV infection by age groupS3 Table. Average costs (per patient) and total cost of medical care of attention for patients with asymptomatic non-icteric HAV infection (year 2019).S4 Table. Average costs (per patient) and total cost of medical care of attention for patients with symptomatic icteric HAV infection (year 2019).S5 Table. Average costs (per patient) and total costs of medical care of attention for patients with HAV infection + FHF or ALF (year 2019).S6 Table. Total cost of medical care of attention for patients with LT due to FHF or ALF secondary to HAV infection (year 2019).S1 Fig. Incidence rate for diagnosed/detected HAV cases by sex and year in Mexico.S2 Fig. Incidence rate for diagnosed/detected HAV cases by region and year in Mexico.S3 Fig. Total sum of patients hospitalized for HAV infection by state in Mexico throughout the observation period (2000–2019).S4 Fig. Total sum of patients hospitalized due to HAV + FHF or ALF by state in Mexico throughout the observation period (2000–2019).S5 Fig. Total sum of patients with LT due to FHF or ALF by state in Mexico throughout the observation period (2007–2019).S6 Fig. Total sum of deaths due to HAV infection by state in Mexico throughout the observation period (2000–2019).
(DOCX)
