# Supplementary material for: Burden of disease and associated complications of hepatitis a in children and adults in Mexico: A retrospective database study
Source: PLoS One. 2022 May 18;17(5):e0268469. doi: 10.1371/journal.pone.0268469 (PMC9116942; doi:10.1371/journal.pone.0268469)
Supplement: S1 File — S1 Table. Overview of study sources. S2 Table. Hospitalized rate (%) comparison for HAV infection by age groupS3 Table. Average costs (per patient) and total cost of medical care of attention for patients with asymptomatic non-icteric HAV infection (year 2019).S4 Table. Average costs (per patient) and total cost of medical care of attention for patients with symptomatic icteric HAV infection (year 2019).S5 Table. Average costs (per patient) and total costs of medical care of attention for patients with HAV infection + FHF or ALF (year 2019).S6 Table. Total cost of medical care of attention for patients with LT due to FHF or ALF secondary to HAV infection (year 2019).S1 Fig. Incidence rate for diagnosed/detected HAV cases by sex and year in Mexico.S2 Fig. Incidence rate for diagnosed/detected HAV cases by region and year in Mexico.S3 Fig. Total sum of patients hospitalized for HAV infection by state in Mexico throughout the observation period (2000–2019).S4 Fig. Total sum of patients hospitalized due to HAV + FHF or ALF by state in Mexico throughout the observation period (2000–2019).S5 Fig. Total sum of patients with LT due to FHF or ALF by state in Mexico throughout the observation period (2007–2019).S6 Fig. Total sum of deaths due to HAV infection by state in Mexico throughout the observation period (2000–2019). (DOCX) [file pone.0268469.s001.docx]

# Supporting information

**S1 Table. Overview of study sources.**

| **Databases** | **Type of data** |
| --- | --- |
| CONAPO [22] | Official population estimates for the target age groups (ages [YoA] < 1, 1–4, 5–9, 10–14, 15–19, 20–24, 25–44, 45–49, 50–59, 60–64, ≥ 65) |
| SINAVE (DGE/DGAE) [23, 24] | Morbidity and mortality related to HAV infection (ICD-10 codes [21]) |
| Statistical Yearbooks [25-27] | Morbidity and mortality related to HAV infection and its complications |
| SINAIS (DGIS) [28] | Morbidity and mortality related to HAV infection (ICD-10 codes [21]) |
|  | Morbidity and mortality related to HAV infection complications (ICD-10 codes [21]) |
| INAI [29] | Morbidity and mortality related to HAV infection and its complications (ICD-10 codes [21]) |
| INAI [29] /CENATRA [30] | Liver transplant due to HAV infection |
| IMSS / DOF [31] | Unitary costs/tariffs for medical direct costs estimation |
| GRD IMSS [32] |  |
| COMPRANET 5.0 [33] |  |

CENATRA: Centro Nacional de Trasplantes; CONAPO: Consejo Nacional de Población; DGE: Dirección General de Epidemiologia; DGAE: Dirección General Adjunta de Epidemiologia; DGIS: Dirección General de Información en Salud; HAV: hepatitis A virus; GRD IMSS: Grupos Relacionados con el Diagnóstico (GRD’s) del IMSS; ICD-10: International Statistical Classification of Diseases and Related Health Problems, Tenth Revision; IMSS/DOF: Costos Unitarios por Nivel de Atención Médica del IMSS; INAI: Instituto Nacional de Transparencia, Acceso a la Información y Protección de Datos Personale; SINAIS: Sistema Nacional de Información en Salud; SINAVE: Sistema Nacional de Vigilancia Epidemiológica; YoA: years of age.

**S2 Table. Hospitalized rate (%) comparison for HAV infection by age group.**

| Age Group | Diagnosed/detected patients due to HAV Infection | | | Hospitalized Patients due to  HAV Infection | | | (%) Hospitalization  Rate due to  HAV Infection* | | | (%) Hospitalization Rate Comparison  (2019 vs year) ** | |
| --- | --- | --- | --- | --- | --- | --- | --- | --- | --- | --- | --- |
|  | **2000** | **2010** | **2019** | **2000** | **2010** | **2019** | **2000** | **2010** | **2019** | **2000** | **2010** |
| < 1 | 255 | 97 | 32 | 58 | 16 | 13 | 22.7% | 16.5% | 40.6% | 78.6% | 146.3% |
| 1 - 9 | 14,237 | 11,129 | 4,372 | 323 | 380 | 260 | 2.3% | 3.4% | 5.9% | 162.1% | 74.2% |
| 10 - 19 | 4,346 | 4,594 | 3,136 | 139 | 321 | 254 | 3.2% | 7.0% | 8.1% | 153.2% | 15.9% |
| 20 - 64 | 2,266 | 2,444 | 2,459 | 81 | 290 | 367 | 3.6% | 11.9% | 14.9% | 317.5% | 25.8% |
| ≥ 65 | 127 | 127 | 76 | 19 | 25 | 23 | 15.0% | 19.7% | 30.3% | 102.3% | 53.7% |
| Total | **21,231** | **18,391** | **10,075** | **620** | **1,032** | **917** | **2.9%** | **5.6%** | **9.1%** | **211.7%** | **62.2%** |

**(%) Hospitalization rate due to HAV infection:* hospitalized patients due to HAV infection/diagnosed-detected patient due to HAV infection.

***(%) Hospitalization rate comparison due to HAV infection:* (%) Hospitalization rate due to HAV infection in 2019 in specific age group – (%) Hospitalization rate due to HAV infection in a specific year and age group/(%) Hospitalization rate due to HAV infection in a specific year and age group.

**S3 Table. Average costs (per patient) and total cost of medical care of attention for patients with asymptomatic non-icteric HAV infection (year 2019).**

| **Age**  **group (YoA)** | **Asymptomatic non-icteric HAV infection patients** | **Cost per patient^a^**  **($ MXN**  **year 2019)** | **Total cost**  **($ MXN**  **year 2019)** |
| --- | --- | --- | --- |
| < 1 | 3 | 11,809.4 | 35,428.3 |
| 1–9 | 444 | 11,810.1 | 5,243,678.7 |
| 10–19 | 319 | 11,810.2 | 3,767,468.3 |
| 20–64 | 248 | 13,573.4 | 3,366,192.0 |
| ≥ 65 | 8 | 13,317.4 | 106,539.3 |
| Total | 1,022 | - | 12,519,306.6 |

HAV: hepatitis A virus; $ MXN: Mexican peso; YoA: years of age.

^a^ Includes: costs related to diagnosis and outpatient management (consultations, laboratory, imaging studies).

**S4 Table. Average costs (per patient) and total cost of medical care of attention for patients with symptomatic icteric HAV infection (year 2019).**

| **Age group (YoA)** | **Symptomatic (icteric) HAV infection patients** | **Cost per patient^a^**  **($ MXN**  **year 2019)** | **Total cost**  **($ MXN year 2019)** |
| --- | --- | --- | --- |
| < 1 | 29 | 45,389.4 | 1,316,293.9 |
| 1–9 | 3,924 | 40,089.0 | 157,309,401.6 |
| 10–19 | 2,814 | 40,606.5 | 114,266,552.7 |
| 20–64 | 2,193 | 40,900.2 | 89,694,191.3 |
| ≥ 65 | 66 | 42,292.4 | 2,791,296.1 |
| Total | 9,026 | - | 365,377,735.6 |

HAV: hepatitis A virus; $ MXN: Mexican peso; YoA: years of age.

^a^ Includes: costs related to diagnosis, hospitalization, and outpatient management (consultations, laboratory, imaging studies).

**S5 Table.** **Average costs (per patient) and total costs of medical care of attention for patients with HAV infection + FHF or ALF (year 2019).**

| **Age group (YoA)** | **HAV infection + FHF/ALF patients** | **Cost per patient^a^**  **($ MXN**  **year 2019)** | **Total cost**  **($ MXN year 2019)** |
| --- | --- | --- | --- |
| < 1 | 0 | 68,604.5 | 0.0 |
| 1–9 | 4 | 109,919.5 | 439,678.2 |
| 10–19 | 2 | 105,535.7 | 211,071.5 |
| 20–64 | 18 | 152,377.0 | 2,742,785.7 |
| ≥ 65 | 2 | 154,883.6 | 309,767.1 |
| Total | 26 | - | 3,703,302.5 |

ALF: acute liver failure; HAV: hepatitis A virus; FHF: fulminant hepatic failure; $ MXN: Mexican peso; YoA: years of age.

^a^ Includes: costs related to diagnosis, hospitalization, and outpatient management (consultations, laboratory, imaging studies).

**S6 Table. Total cost of medical care of attention for patients with LT due to FHF or ALF secondary to HAV infection (year 2019).**

| **Age group (YoA)** | **Patients with LT due to FHF or ALF secondary to HAV infection** | **Cost per patient**  **(GRD´s**  **year 2019,**  **$ MXN)^a^** | **Total cost**  **($ MXN year 2019)** |
| --- | --- | --- | --- |
| < 1 | 0 | 1,262,232.6 | 0.0 |
| 1–9 | 0 |  | 0.0 |
| 10–19 | 0 |  | 0.0 |
| 20–64 | 1 |  | 1,262,232.6 |
| ≥ 65 | 0 |  | 0.0 |
| Total | 1 | - | 1,262,233.6 |

ALF: acute liver failure; HAV: hepatitis A virus; FHF: fulminant hepatic failure; GRD: Grupos Relacionados con el Diagnóstico; LT: liver transplant; $ MXN: Mexican peso; YoA: years of age.

^a^ Average costs between GRD 005 (liver transplant with medical complications or intestinal transplant) and GRD 006 (liver transplant without medical complications). Inflation Factor used 1.0283 (Jan 2019–Dec 2019).

**S1 Fig. Incidence rate for diagnosed/detected HAV cases by sex and year in Mexico.**

**
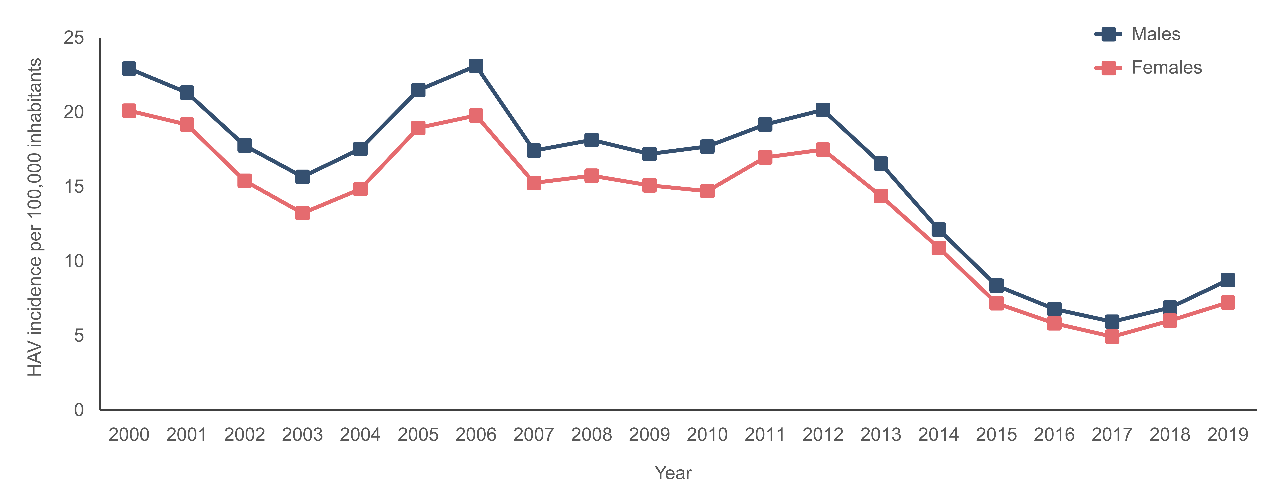
**

HAV: hepatitis A virus.

**S2 Fig. Incidence rate for diagnosed/detected HAV cases by region and year in Mexico.**

**
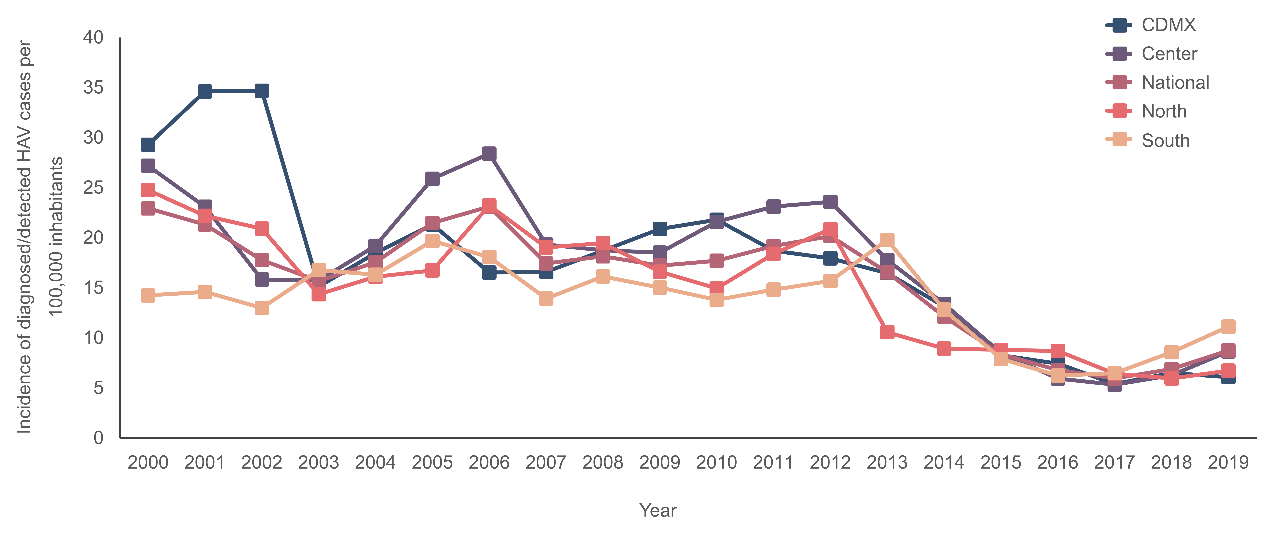
**

HAV: hepatitis A virus.

**S3 Fig. Total sum of patients hospitalized for HAV infection by state in Mexico throughout the observation period (2000–2019).**


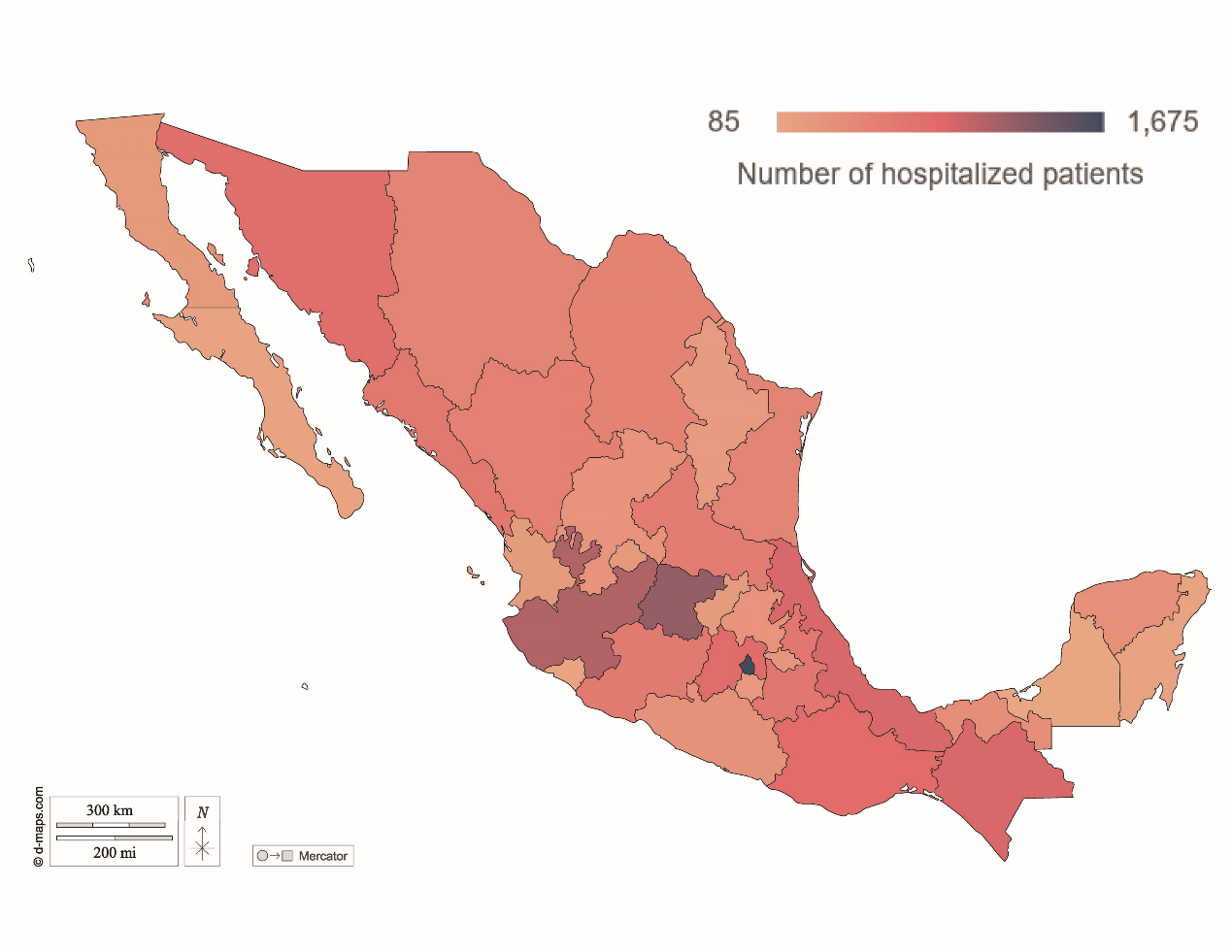


HAV: hepatitis A virus. Map adapted from <https://d-maps.com/pays.php?num_pay=288&lang=en> with permission.

**S4 Fig. Total sum of patients hospitalized due to HAV + FHF or ALF by state in Mexico throughout the observation period (2000–2019).**


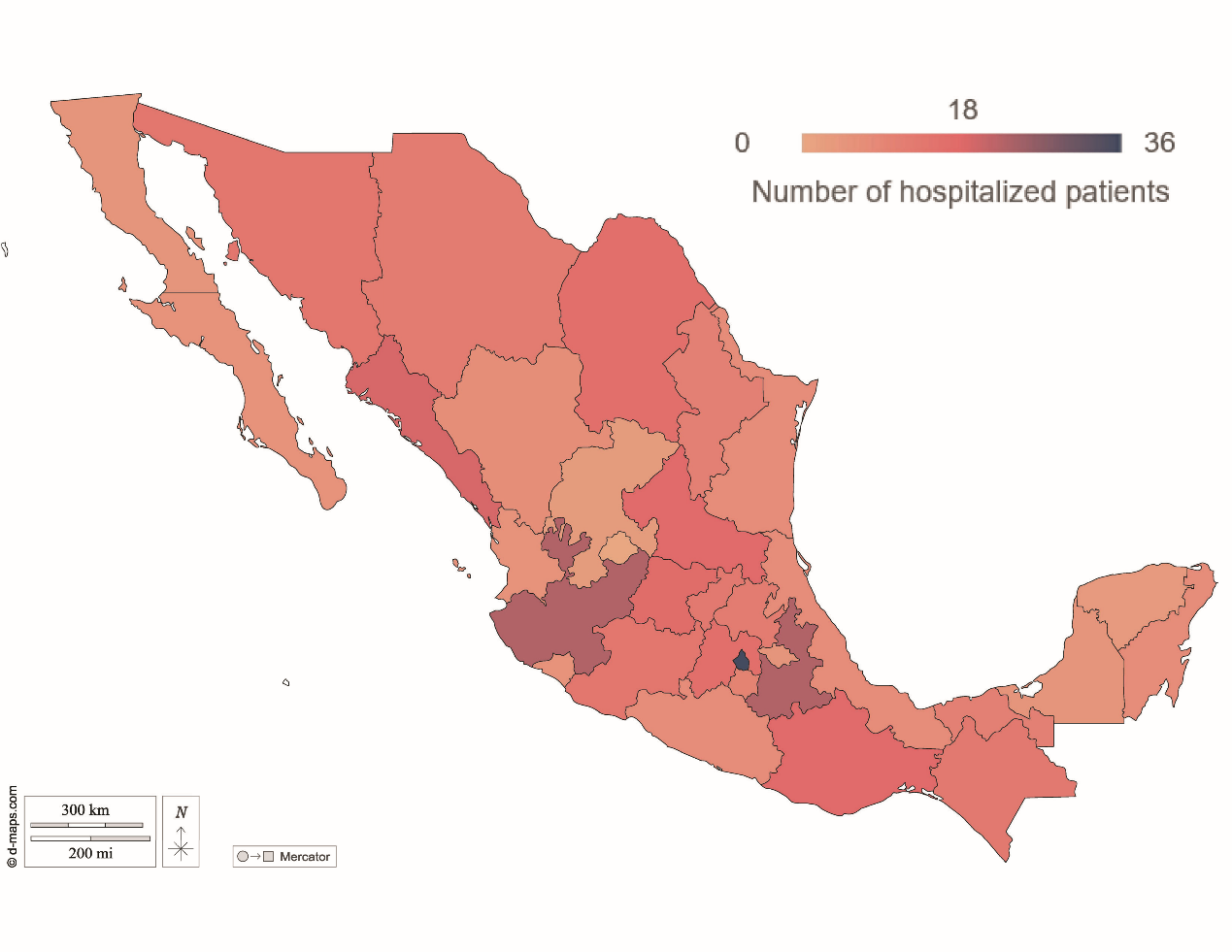


ALF: acute liver failure; HAV: hepatitis A virus; FHF: fulminant hepatic failure. Map adapted from <https://d-maps.com/pays.php?num_pay=288&lang=en> with permission.

**S5 Fig. Total sum of patients with LT due to FHF or ALF by state in Mexico throughout the observation period (2007–2019).**

**
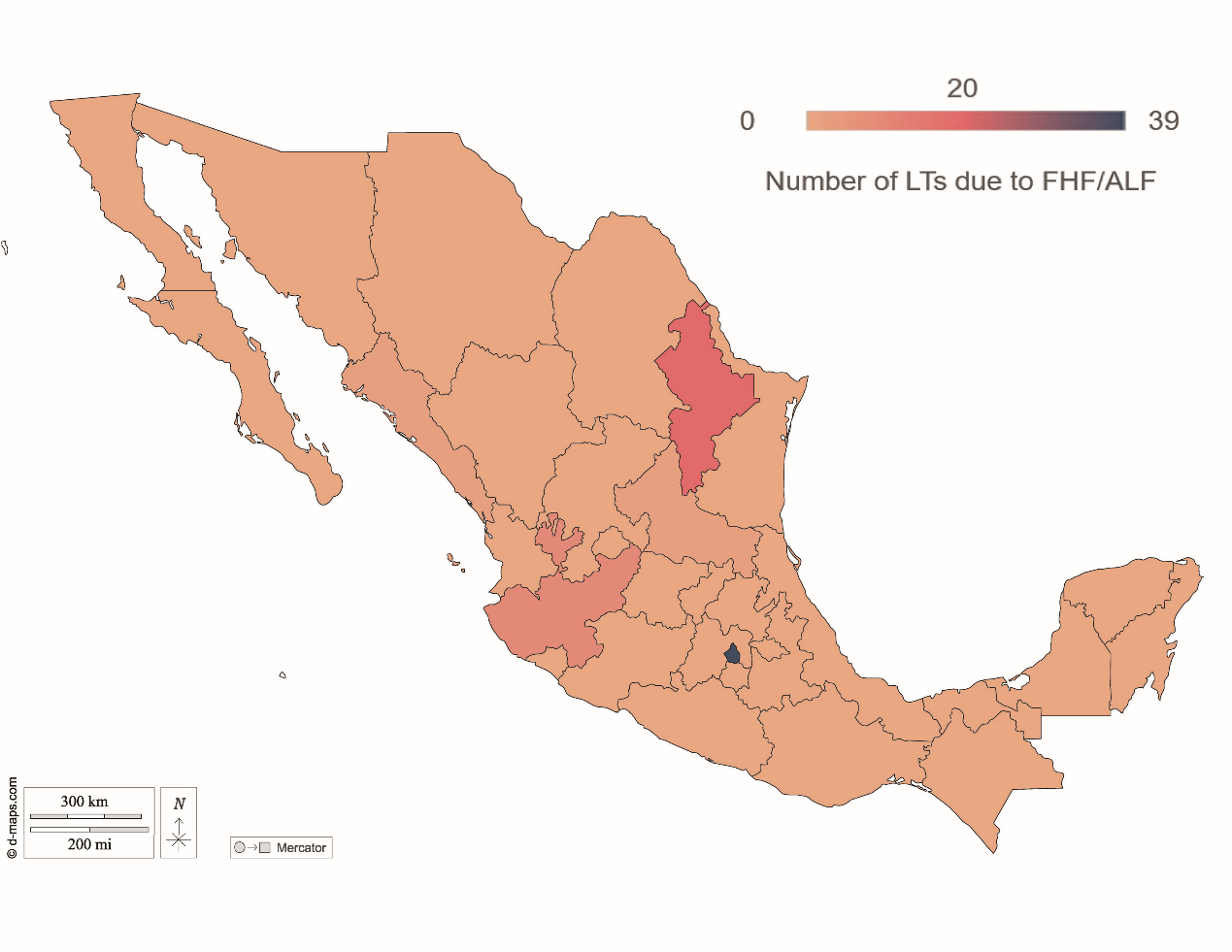
**

ALF: acute liver failure; FHF: fulminant hepatic failure; LT: liver transplant. Map adapted from <https://d-maps.com/pays.php?num_pay=288&lang=en> with permission.

**S6 Fig. Total sum of deaths due to HAV infection by state in Mexico throughout the observation period (2000–2019).**


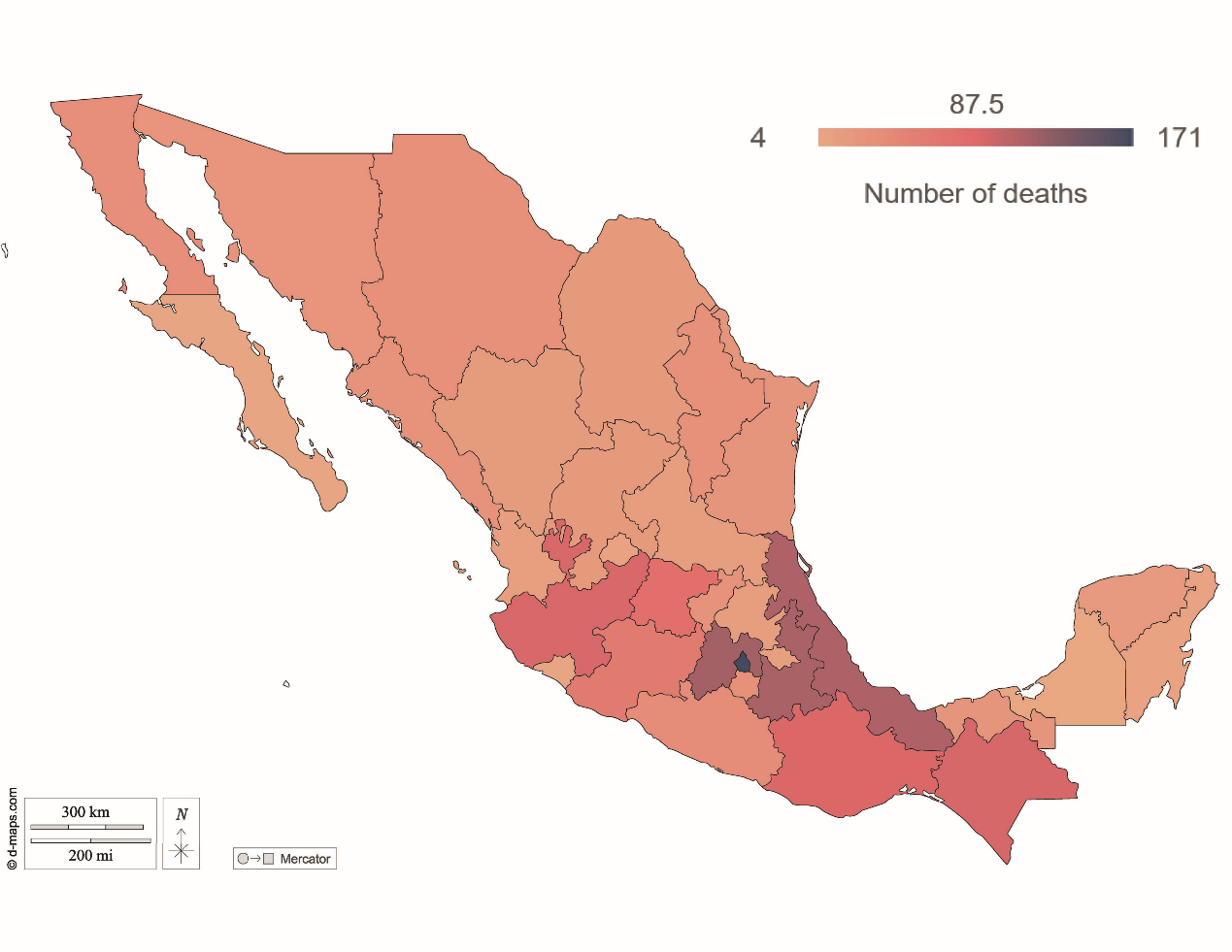


HAV: hepatitis A virus. Map adapted from <https://d-maps.com/pays.php?num_pay=288&lang=en> with permission.
